# Perceptions, experiences and expectations of Iraqi medical students

**DOI:** 10.1186/s12909-018-1156-8

**Published:** 2018-03-27

**Authors:** Riyadh Lafta, Waleed Al-Ani, Saba Dhiaa, Megan Cherewick, Amy Hagopian, Gilbert Burnham

**Affiliations:** 1 Al Mustansiriya University, College of Medicine, Baghdad, Iraq; 2 Department of Community Health, College of Health and Medical Technology, Baghdad, Iraq; 3 The Johns Hopkins Bloomberg School of Public Health, Baltimore, MD USA; 4 University of Washington, School of Public Health, Seattle, WA USA

**Keywords:** Iraq, Medical students, Medical education, Career choices, Immigration

## Abstract

**Background:**

The environment for medical education in Iraq has been difficult for many years. The 2003 invasion of Iraq accelerated a steady emigration of faculty and graduates. Kidnappings and deaths of doctors became commonplace. To understand current career plans, expectations and perceptions of medical students, three Baghdad medical schools were surveyed.

**Methods:**

Written questionnaires were completed by 418 medical students variously in their 4th, 5th and 6th (final)years of training. We asked about perceptions of the quality of their medical education, the quality of health services in Iraq generally, and about deaths, injuries and migration of faculty, classmates and family.

**Results:**

The average age of students was 22 years, with 59% women. Most students (90%) were originally from Baghdad. Although there were some positive responses, many students (59%) rated the overall quality of their medical education as fair or poor. Three-fourths of students believed the quality of hospital care in Iraq to be only fair or poor. A majority of students (57%) stated they were thinking frequently or all the time about leaving Iraq after graduation. Reasons given for leaving included the desire for further education, seeking a better lifestyle and fleeing conflict. Leading reasons for staying included the pull of friends and family, familiarity with the health system, and a sense of responsibility to the country. Nearly one in five (18%) students reported the death of a family member attributable to intentional violence, and 15% reported the violent death of a medical school classmate or faculty member since the 2003 invasion. Half the students reported at least one school faculty members had left Iraq because of the war.

**Conclusion:**

Medical students hold a mediocre view of the quality of their medical education and of Iraq’s health system. Many of their faculty members have left the country. The majority of students may leave Iraq after graduation, afforded the opportunity. This poses a significant problem for staffing an already demoralized and stressed health system. Current circumstances suggest the situation will continue to deteriorate.

**Electronic supplementary material:**

The online version of this article (10.1186/s12909-018-1156-8) contains supplementary material, which is available to authorized users.

## Background and rationale

Armed conflict has a profound effect on health services. Health facilities are damaged, medicines and supplies are inadequate, communications and transport are interrupted, and yet it is perhaps the loss of professional staff that does most to undermines health services. Conflict has driven medical staff to seek employment outside of their home countries, as has been the history in Cuba, Lebanon, Iran, and Liberia [1–4]. However, the consequences of protracted conflict on medical education has received little attention. During the Liberian civil war, damage to the country’s single medical school caused faculty to flee and reduced the 2007 graduating class to four from the usual 40 [5]. In Croatia, medical students at the University of Zagreb managed to continue their studies, but assumed additional conflict-related duties [6]. With the Islamic revolution in Iran, many faculty left and the medical curriculum was altered to include non-medical topics [4].

In the first half-dozen years following the 2003 U.S.-led invasion of Iraq, an estimated 2000 doctors were killed, more than 250 were kidnapped, and 12% of the country’s hospitals were destroyed [7]. Violent deaths among doctors in Baghdad occurred at an annual rate of nearly 50 per 1000 at the peak of the conflict [8]. In 2006, annual physician emigration neared 300 per 1000 per year, and by 2008, roughly 9000 doctors and 15,000 nurses remained to care for a population of 28 million [8, 9]. By 2017 the number of doctors had increaserd somewhat to 8.4 per 10,000 population. Surveys of Iraqi physicians have found more than half of those who left the country did so for security reasons, while more than half of those remaining were seeking alternative employment or planning to leave the country soon, often citing similar concerns for their safety [10, 11]. A recent survey found that the majority of medical students also plan to leave Iraq after graduation, but few studies have examined the underlying reasons [12]. A set of interviews with eight medical school deans and 197 students from various medical schools in Iraq found that the continuing conflict had a substantial effect on medical education and attitudes of medical students [12]. Overall, Iraq experienced a 50% reduction in its medical workforce during the U.S. occupation despite government measures to offer better working conditions and improve security [7, 8]. The 2014 invasion of Iraq by ISIS (Islamic State of Iraq and Syria) and ongoing turmoil continues to take a toll on the country’s health services [13].

During the rule of Saddam Hussein, many medical doctors graduated with inadequate training and low motivation [14]. The intellectual embargo against Iraq significantly undermined medical education in Iraq [15]. In the Saddam years it was difficult for doctors to immigrate, and sometimes their diplomas were withheld by authorities to prevent flight. The violence that followed the American-led invasion in 2003 caused many doctors to flee. Interviews with refugee Iraqi doctors in Jordan in 2006, found half of doctors reported only a third of positions for medical officers in their former facilities in Iraq were filled. Estimates suggest perhaps half of Iraqi doctors fled the country or were killed during the 13 years of protracted conflict in Iraq [7]. The Nineveh College of Medicine, once the site of extensive specialty training, was closed after the seizure of Mosul by ISIS [16]. While Iraqi doctors have continued to go abroad, many others migrate to less conflict-affected parts of the country [17].

After World War I, the League of Nations gave Great Britain mandates for three former Ottoman provinces which were united to form modern-day Iraq [18]. Iraq’s first medical school was founded in Baghdad in 1927, with Sir Harry Sinderson as its first dean [19]. Gradually medical education expanded to reach its current number of 25 medical schools, of which 23 are listed in the World Directory of Medical Schools [20]. The number of medical schools has doubled since 2005 [21]. The 6-year undergraduate medical curriculum is taught in English. The curricular content follows British conventions, although there have been recent calls for updating the curricula and teaching methods [22]. Medical education in Iraq is free, as are required textbooks. In the past, graduates seeking specialty training would travel abroad, usually to Great Britain, to secure specialty qualifications before returning to Iraq. Once medical education and clinical care in Iraq had the reputation of being the best in the region [16].. Today, medical education in Iraq is held back by a lack of facilities, limited financial support, loss of experienced faculty, and a static traditional medical curriculum urgently in need of revitalization [23].

With the retirement, death, and migration of many of Iraq’s medical practitioners, concerns have been raised about future provision of medical care for Iraq‘s 38 million people. The intent of this study was to assess the professional and personal plans of physicians in training in Iraq. Such information could guide Iraqi health services planning medical officer staffing levels. The students in this study have known only conflict during the past 13 years.

## Methods

To better understand perceptions, experience and future plans of medical students in their final 3 years of university, we carried out this study of 418 medical students in the three medical schools in Baghdad in 2015.

Questions were developed by researchers with experience studying health services and health training requirements in Iraq. The questionnaire was based, in part, on surveys developed for other clinical trainees in settings of stress [24, 25]. The final self-administered paper questionnaire consisted of 28 questions, requiring 25–30 min to complete. It was given to 204 fourth year, 128 fifth year and 86 sixth (final) year medical students in Baghdad during late 2015. Respondents included 119 medical students from the University of Baghdad, 99 from Al-Kindy University and 200 from Al-Mustansiriya University. The questionnaire is available as a separate file formatted in Word, entitled Baghdad Medical Student Questionnaire (Additional file 1).

This study received ethical approval from the Scientific Committee of the College of Medicine, Al Mustansiriya University in Baghdad, and analysis of unlinked data was declared as not human subjects research by the Johns Hopkins Bloomberg School of Public Health and the University of Washington. The nature of the study and its intent were discussed with the three medical school deans, and their permission obtained. Before the questionnaire was administered, it purpose was explained to students and their individual permission secured. Names of the students were not recorded. Data were computer entered in Baghdad with SPSS. Analysis was conducted in Baghdad and Baltimore using Stata on datasets without unique identifiers.

## Results

Students completing the questionnaire were those present in the respective university at the time of the survey and not away on clinical attachments at other sites. All students physically present completed the questionnaires. Medical student respondents ranged in age from 20 to 28 years, with a mean age of 21.9 years (Tables 1 and 2). More than half (59%) were female. Two-thirds of students reported a relative who was a medical doctor, but only 12% reported a doctor or dentist relative who was living outside Iraq. When disaggregated by year, the 6th year students did not differ substantially from 4th and 5th year students in the proportion with family who were doctors. There were also no differences among classes or medical schools in the presence of family members outside Iraq, or students with family members outside Iraq who were doctors or dentists (data not shown). The existence of expatriate family members was not associated with expressed desired to leave Iraq after graduation. Most of the medical students included in this survey were from Baghdad (90%), with the balance coming from other governorates.

**Table 1:** Baghdad medical student characteristics

	Number	Percent
Age		
Age 20–22	306	73.2
Age 23–24	93	22.3
Age 25–28	19	4.5
*Total*	*418*	*100*
Mean Age (SD)	21.9 (1.3)	
Sex		
Male	171	40.9
Female	247	59.1
Marital Status		
Married	5	1.2
Single	413	98.8
Home before medical school		
Baghdad	374	89.5
Other cities in Iraq	34	8.1
Outside Iraq	10	2.4
Have family living outside of Iraq		
Yes	104	24.9
No	314	75.1
Has a relative who is a doctor		
Yes	275	65.8
No	143	34.2
Has a relative who is a doctor or dentist and living outside of Iraq		
Yes	51	12.2
No	367	87.8
*Total*	*418*	*100*

Data source is a 2015 survey of 418 medical students from three medical schools in Baghdad

**Table 2:** Baghdad medical student level of agreement with statements about aspects of their medical training

	Strongly disagree N (%)	Somewhat disagree N (%)	Somewhat agree N (%)	Strongly agree N (%)	Not sure N (%)
Clinical rotations are well organized to help me learn the topic	61 (14.6)	78 (18.7)	159 (38.0)	65 (15.6)	55 (13.2)
Access to current textbooks and journals is generally good	73 (17.5)	76 (18.2)	147 (35.2)	64 (15.3)	58 (13.9)
Faculty conduct teaching sessions when scheduled	57 (13.6)	79 (18.9)	177 (42.3)	60 (14.4)	45 (10.8)
Faculty keep up to date in the latest developments in their field	56 (13.4)	99 (23.7)	149 (35.7)	59 (14.1)	55 (13.2)
Faculty are generally good teachers with a strong interest in helping us learn	56 (13.4)	85 (20.3)	180 (43.1)	54 (12.9)	43 (10.3)
Faculty private practice responsibilities interferes with teaching responsibilities	87 (20.8)	95 (22.7)	114 (27.3)	48 (11.5)	74 (17.7)
The basic science curricula were well organized to help me learn	74 (17.7)	116 (27.8)	135 (32.3)	30 (7.2)	63 (15.1)
Faculty knowledge and dedication to teaching is very high	107 (25.6)	87 (20.8)	147 (35.2)	17 (4.1)	60 (14.4)
	Poor	Fair	Good	Excellent	
Overall perceived quality of medical training	64 (15.3)	184 (44.0)	162 (38.8)	8 (1.9)	

Data source is a 2015 survey of 418 medical students from three medical schools in Baghdad

Very few of the medical students queried (*n* = 8, 2%) rated their medical training as excellent, and fewer than half (*n* = 162, 39%) said it was good; most said it was fair (44%) or poor (15%). Those indicating “not sure” were excluded. The strongest positive opinions of medical training had to do with the quality of faculty, with 234 (62%) reporting faculty were generally good teachers and had a strong interest in helping students learn, and another 64% reporting faculty conduct teaching sessions when scheduled. There were 62% who said clinical rotations were well organized. In somewhat of a contradiction, 54% of students said they didn’t think faculty knowledge or dedication to teaching was very high. The strongest negative student views were about access to journals and textbooks (41%), faculty keeping up to date with medical developments (43%), and the organization of the basic science curriculum (54%). Over half (53%) of students felt that to some degree faculty private practice responsibilities interfered with their teaching responsibilities. Differences in perceptions about various aspects of medical education did not differ substantially among students in the three medical schools.

Medical students were not very enthusiastic about the quality of medical care in Iraq, with fewer than one in ten saying the quality of care was excellent (Table 3). Only for private clinics did more than half of medical students with opinions characterize the quality of care good or excellent (218 or 55%, not including “don’t know” responses). Overall care at public hospitals was thought to be good or excellent by 102 (25%), but three quarters (300, or 75%) said it was only fair or poor. Quality of care in public primary health care clinics was rated only marginally better than hospitals. One in five students said current working conditions for doctors were good or excellent (*n* = 74, 19%), with 86% worried about personal security conditions for doctors. The attitude of patients and family attitudes toward doctors was rated as excellent or good by only 84 medical students (21%).

**Table 3:** Baghdad medical student opinions about health care in Iraq

	Excellent N (%)	Good N (%)	Fair N (%)	Poor N (%)	Don’t know N (%)	Total N (%)
Quality of health care received at private clinics	51 (12.2)	167 (40.0)	126 (30.1)	56 (13.4)	18 (4.3)	418 (100)
Quality of health care received at hospitals	32 (7.7)	70 (16.8)	120 (28.7)	180 (43.1)	16 (3.8)	418 (100)
Attitude and concern of doctors for patients	31 (7.4)	102 (24.4)	161 (38.5)	99 (23.7)	25 (6.0)	418 (100)
Salary and Income for doctors	31 (7.4)	92 (22.0)	124 (29.7)	141 (33.7)	30 (7.2)	418 (100)
Availability of laboratory tests when needed	25 (6.0)	124 (29.7)	151 (36.1)	90 (21.5)	28 (6.7)	418 (100)
Attitudes and concern of other health care workers for patients	23 (5.5)	76 (18.2)	155 (37.1)	133 (31.8)	31 (7.4)	418 (100)
Quality of health care received at primary health care clinics	21 (5.0)	96 (23.0)	141 (33.7)	128 (30.6)	32 (7.7)	418 (100)
Availability of medicines and supplies when needed	19 (4.6)	115 (27.5)	126 (30.1)	132 (31.6)	26 (6.2)	418 (100)
Attitude of patients and families toward doctors	18 (4.3)	66 (15.8)	87 (20.8)	221 (52.9)	26 (6.2)	418 (100)
Safety and security of doctors	18 (4.3)	38 (9.1)	53 (12.7)	287 (68.7)	22 (5.3)	418 (100)
Working Conditions for doctors	10 (2.4)	64 (15.3)	93 (22.3)	223 (53.4)	28 (6.7)	418 (100)

Data source is a 2015 survey of 418 medical students from three medical schools in Baghdad

Students were asked about further training after medical school. By their fourth year and beyond, and there were 61% who said they were starting to form specialty preferences. Surgery and surgical specialties were the most popular, being considered by 83 (20%) followed by 55 (13%) for training in internal medicine or the medical specialties. Other popular choices were pediatrics, 29 (7%), dermatology, 27 (7%), obstetrics and gynecology 24 (6%) and radiology 16 (4%). Only 7 (2%) would choose family medicine. When asked where they would like to complete their specialty training, less than a third (*n* = 121, 29%) said they would chose to do their training in Iraq. Of the 279 (71%) who indicated they were thinking of training outside Iraq, only 64 (15.3%) had a specific location in mind. Although 6th year students thought more about leaving Iraq for further training than 4th years students, the trend was not statistically significant (data not shown).

Asked about where they would choose to practice medicine after their further training, more than half (*n* = 238, 57%) said they thought about leaving Iraq after graduation either frequently or all the time. Another third (136, 33%) said they were considering it occasionally. Only 44 students (11%) said they were not considering leaving Iraq. Despite their preferences for leaving, if they stayed in Iraq, 118 students (28%) said they were very likely to work in Baghdad, and 227 (54%) thought it somewhat likely. When asked about working elsewhere in Iraq, 162 (39%) would definitely not consider this while 189 (45%) would possibly consider working outside Baghdad. For preferred locations for work outside Iraq, the first was Europe, then North America, elsewhere in the Middle East, or Australia/New Zealand (data not shown).

Leading the list of reasons for wishing to stay in Iraq was connections with families and friends with 342 (82%) responses, followed by feelings of responsibility to assist Iraq (58%) and familiarity with the health services (55%) (Table 4). By comparison, the leading reasons given to leave Iraq after graduation were the anticipation of a better life style (85%) the opportunities for advance training (84%), and a flight from continuing conflict (82%).

**Table 4:** Motivation for Staying or Leaving Iraq

	Most important N (%)	Somewhat important N (%)	Not very important N (%)	Least important N (%)	Total N (%)
Motivation for Staying in Iraq					
To be with family and friends	226 (54.1)	116 (27.8)	36 (8.6)	40 (9.6)	418 (100)
Familiar with health care system in Iraq	104 (24.9)	124 (29.7)	71 (17.0)	119 (28.5)	418 (100)
Feel responsibility to help my country	97 (23.2)	144 (34.5)	80 (19.1)	97 (23.2)	418 (100)
Positions I can get here are better than I can get outside of Iraq	64 (15.3)	127 (30.4)	85 (20.3)	142 (34.0)	418 (100)
Personal life style in Iraq is what I like	57 (13.6)	108 (25.8)	71 (17.0)	182 (43.5)	418 (100)
Motivation for Leaving Iraq					
Better personal lifestyle	260 (62.2)	95 (22.7)	39 (9.3)	24 (5.7)	418 (100)
Avoid war and conflict	253 (60.5)	89 (21.3)	40 (9.6)	36 (8.6)	418 (100)
Seek advanced training	242 (57.9)	107 (25.6)	31 (7.4)	38 (9.1)	418 (100)
Better professional opportunities	221 (52.9)	122 (29.2)	43 (10.3)	32 (7.7)	418 (100)
Better pay and working conditions	151 (36.1)	161 (38.5)	70 (16.8)	36 (8.6)	418 (100)
To be with family	143 (32.3)	119 (28.5)	59 (14.1)	97 (23.2)	418 (100)

Data source is a 2015 survey of 418 medical students from three medical schools in Baghdad

Nearly three in four students said political and armed conflict in Iraq had influenced their choice of a future career. In all, 300 of the 418 students indicated this was a very important or somewhat important factor in their career choices (data not shown). Analysis by trend showed this concern was greater among 5th and 6th year students than for 4th year students (*X*^2^ = 15.2, *p =* 0.0001) (data not shown).

The majority of respondents said desirability and professional satisfaction from a medical career in Iraq had deteriorated during the past 3 years. When these data were disaggregated by student class, desirability of a medical practice Iraq were poorer among the 6th year students than among 4th years students (*X*^*2*^ = 12.76, *p* < 0.001). Although expectations of professional satisfaction from medical practice and having a satisfying personal life in Iraq were overall low, there was no significance difference among the fourth, fifth and sixth year students (Table 5).

**Table 5:** Changes in medical profession desirability in the past 3 years as ranked by Baghdad medical students

	Improved N (%)	Stayed the same N (%)	Deteriorated N (%)	Not Sure N (%)	Total N (%)
Desirability to practice medicine in Iraq	47 (11.2)	109 (26.1)	222 (53.1)	40 (9.6)	418 (100)
Professional satisfaction from the practice of medicine in Iraq	29 (6.9)	136 (32.5)	200 (47.9)	53 (12.7)	418 (100)
Potential of living a satisfying personal life while practicing preferred specialty in Iraq	21 (5.0)	88 (21.1)	268 (64.1)	41 (9.8)	418 (100)

Data source is a 2015 survey of 418 medical students from three medical schools in Baghdad

Nearly one in five students (*n* = 73, 18%) reported a death in the immediate family since the invasion of Iraq in 2003, and for 35 students, more than one family member was killed. Fifty students reported the serious injury of a family member by conflict since 2003, and 27 students reported the serious injury of more than one family member. Serious injuries were more common among families of 6th year students (OR = 3.10) than 4th or 5th years students (*X*^2^ = 8.93. *p =* 0.002).

Some 62 (15%) students reported the death of a classmate or medical school faculty from violent conflict, and 32 (8%) reported more than one had been killed. Half (*n* = 214, 51%) reported a faculty member had left Iraq, while 155 students reported more than one faculty member had left. There were 49 students (12%) who reported five or more of the medical school faculty had left. This is undoubtedly an underestimate, as students would not have been acquainted with all faculty members at their medical school, especially in their pre-clinical years.

## Discussion

The 418 Iraqi students from three Baghdad medical schools in our study had mixed feelings about the quality of their medical studies. While few specific areas of unsatisfactory training were singled out, the general assessment of their education was low. Medical students perceive that personal and professional satisfaction among medical professionals in Iraq has deteriorated. The desire to leave Iraq after graduation for professional and for personal reasons was very strong, and the continuing armed conflict contributes heavily to this.

Our findings from this student survey corroborate other surveys of Iraqi physicians, highlighting the need for improved training, equipment, salaries, nursing staff, and facilities [14, 26, 27]. One study found only 12% of doctors working at Al-Kadhimiya Teaching Hospital expressed a high level of job satisfaction, with the lowest satisfaction factor being pay and the highest was nature of work [28]. In the post-Saddam Hussein era, physician wages increased from US$380 to $5100 annually, still relatively low considering the workload and risk level [29]. Some doctors are threatened because of their current or past associations with the government. Many are targeted for kidnapping since they earn more money than most and thus secure a higher ransom [30, 31].

The loss of faculty has impaired the teaching capacity of Iraq’s medical schools [32]. With the absence of professional staff, much of the pre-clinical laboratory teaching has been reduced to theoretical explanations, without the practical laboratory sessions [16]. Religious proscriptions have restricted the dissection of cadavers. Electronic simulations or very old preserved specimens have been used as substitutes. We found mixed responses from students about the quality of their training. Some area of training, like “faculty knowledge and dedication to teaching,” were viewed positively. Other factors such as access to current journals and books, faculty keeping up to date with current developments, interference in training by faculty private practice and the organization of the basic science curriculum got low ratings. On the whole, only 47% of students rated the quality of their medical education as good or excellent. It could be argued that students do not have a comparison model to make this judgment, however students can assess how well their training is preparing them to practice medicine in Iraq.

Students regarded the quality of health services in Iraq as generally poor, particularly for the public sector, with only slightly more than half rated the private sector as good or excellent. The lowest ratings went to physician working conditions and their safety and security. Students were also dissatisfied with the attitudes of doctors, with 34% saying the concern of doctors for patients was good or excellent. This was somewhat better than the good or excellent concern for patients reported for other health care workers (26%). Attitudes of patients and families towards doctors were rated poorly, with only 21% of students saying doctors were highly regarded by their patients. This contrasts with findings in a 2009 survey where most patients reported satisfaction with health worker encounters, though patient expectations may have been low [33]. Poor attitudes of patients is consistent with the reports of hostility and even physical violence toward doctors by patients and families, particularly in the instances of poor clinical outcomes [16, 34, 35].

The deans of medical schools seem to agree with our findings, as reflected in the 2016 survey by Barnett-Vanes et al. [12] Five of eight surveyed medical school deans believed conflict has impaired medical student attainment and training. Deans named missed days of classes, graduation with gaps in knowledge, and poor security in the surrounding region. There were also difficulty with faculty and staff recruitment and retention, finances, email, and internet access [32]. The majority of medical students surveyed in that study also said their training had been impaired by conflict. Reasons included mental exhaustion, personal safety, anxiety, and depression. Despite these challenges, the majority of students in that study had not considered dropping out [12].

Few of the medical students in our study identified family medicine (or other primary care medical specialties) as their career choice. Iraq has an inefficient overly-hospitalized focus to its health services [36]. Even if the Ministry of Health were to follow through on its declared primary health care focus, there would be too few medical officers wishing to work in primary health care facilities to adequately staff these clinics.

The continuing emigration of general practitioners and specialists, added to loss from retirement, will create an increasing number of vacant posts for medical officers. The current density of physicians in Iraq is 8/10,000, about a third of Jordan or Saudi Arabia [37]. At the present time all Iraqi medical graduates can still be assured of job placement in the public sector. If the emigration intentions expressed by medical students are realized, the number of new graduates available to fill vacant posts will be limited. Further, these vacancies are in a health system already perceived to be poorly performing. There should be little expectation that new medical graduates will be highly motivated to work in this system. With a general perception that their own medical education has been mediocre, graduates may not have the medical skills to perform in positions vacated by doctors trained at a time when medical educations standards were higher. The perceived lack of security, which has further deteriorated since this study was conducted in 2015, may only increase the exodus of graduates.

Various avenues can be explored to improve Iraqi medical education. With a trend toward globalization of training, more partnerships and linkages between medical schools in the economically advanced countries and medical schools in Iraq are possible. A common curriculum in English is an advantage. Doctors originally from the Middle East, are increasingly assuming faculty positions in Europe, North American and Australia, and many have established links or could develop these with Iraq schools. In the past few years, the number of free Massive Online Open Courseware (MOOCS) options has increased dramatically, offering the options of blended learning approaches to education in Iraq. However, the critical first step is the willingness of Iraq medical schools to undertake a substantial revision of the curriculum and teaching methods to shift focus to student-centered learning from the traditional lecture-based approach. There have been voices in the past years calling for such a revision in curricular approaches, but so far, the inertia of the current system has discouraged these voices of reform. However, if the country is to retain its graduates, educated at considerable cost to the country, it cannot continue to blame insecurity for their leaving Iraq, but must focus on improving the quality of training and sustain the motivation that originally attracted students to study medicine.

Population studies of civilian mortality in Iraq indicate at least half a million Iraqi deaths have occurred in the first 10 years of conflict [38, 39]. This level of mortality is reflected in reports that nearly one in five (17%) medical students had experienced the death of immediate family member secondary to intentional causes since 2013, and another 50 (12%) students reported intentional injuries among family members.

### Limitations

This survey included only students from Baghdad. It is likely students in other parts of the country could have had differing career plans and perceptions of medical education as well as the functionality of the health services. However, other reports [12, 16] suggest these findings are likely to represent medical students in other Iraq locations as well. Not all medical students in each of the three Baghdad schools were available for the survey on the days this was administered, many being away on clinical attachments. This may have made the survey less representative, though other reports show similar patterns for some of the common questions. The number of 6th year students in our study was smaller than for other years, but their results didn’t differ significantly from students in other years. Perceptions across the three schools didn’t differ significantly, making the inclusions of more students from Al-Mustansiriya than the other two schools less important. There may have been unmeasured factors influencing perceptions reported.

## Conclusions

These findings depict a student population not particularly satisfied with the quality of its medical school training, and who reported decidedly mixed views about the quality of health services in Iraq. When considering the future, the majority are looking to leave Iraq for various reasons. Those who say they are only going abroad for further training are unlikely to return, based on established patterns. There are few reasons to expect young physicians will improve their views of the quality of Iraq’s health care and medical education in coming years. Insufficient numbers of well-trained and motivated medical graduates may portend increasing problems in staffing Iraq’s already troubled health care system. The major reasons graduates might be inclined to stay in Iraq, particularly Baghdad, are the presence of family, familiarity with the Iraqi health system, and feelings of responsibility to contribute to the health of the country. At this time, when Iraq’s oil revenues are at historic lows and extensive military operations are ongoing, there are limited public resources available to improve medical education. Medical graduates choosing to remain in Iraq will enter a dysfunctional and demoralizing health service, when what the country needs is an enthusiastic cohort of young doctors. To reverse the migration and educational issues, a concerted effort to revitalize a static traditional system, replacing this with a strong student-focused approach, perhaps utilizing the many online educational options now available.

## Additional file

Additional file 1: Iraqi Medical Student Questionnaire. (DOC 136 kb)

## Abbreviations

ISIS: Islamic State of Iraq and Syria
MOOCS: Massive Online Open Courseware
N or n: Number
OR: Odds ratio
*p*: Probability
SD: Standard deviation
SPSS: Statistical package for the social sciences
*X*^*2*^: Chi-square
