# Supplementary material for: Perceptions, experiences and expectations of Iraqi medical students
Source: BMC Med Educ. 2018 Mar 27;18:53. doi: 10.1186/s12909-018-1156-8 (PMC5870482; doi:10.1186/s12909-018-1156-8)
Supplement: Supplementary file 1 — Iraqi Medical Student Questionnaire. (DOC 136 kb) [file 12909_2018_1156_MOESM1_ESM.doc]

**Iraqi Medical Student Questionnaire**

#### Consent statement

### Dear Student,

In this survey we want to understand the characteristics of Iraqi medical students and their career intentions. We hope the results of this study will help us better understand how medical students make career choices over the next few years. You may not directly benefit from taking part in this research study. If you choose to be in this voluntary study, please fill out the questionnaire. We will ask you questions about your views of the medical profession in Iraq, your career plans, and, your background. This will take about 10-15 minutes. You do not have to answer every question if you do not choose.

Taking part in this study is voluntary. You can stop at any time. Your responses are anonymous. Your responses are not linked to your name.

If you have any questions or concerns regarding this questionnaire or study, please contact:

Prof Riyadh Lafta, email address [*riyadhlafta@yahoo.com*]

--------------------------------------------------------------------------------------------------------------------

**A. About yourself**

1. Your age ____
2. Name of your medical school
3. Your year in medical school (circle): 4, 5 or 6: _______________
4. Your sex: ***M F***
5. Your marital status (circle one): ***A married B single***
6. Which governorate you lived in before you started university?
7. Your estimated overall ranking in your medical school class *(circle one)*

***A Top 1/3 B Middle 1/3 B Bottom 1/3***

1. Do you have a relative who is a doctor? ***A YES B NO***
2. Do you have a brother, sister, or a parent living outside of Iraq now? ***A YES B NO***
   1. Where (list all locations)? _____________________
   2. Are any of these relatives outside of Iraq a doctor or a dentist? ***A YES B NO***

**B. The quality of your medical education**

1. How would you rate the overall quality of your medical training at this school? *(circle only one)*

**A Excellent B Good C Fair D Poor**

1. How would you rate each of these aspects of your medical training at this school? (check each line)

| **1**=do not agree **2**=mostly disagree **3**=mostly agree  **4**=strongly agree 5=not sure | | | | | | |
| --- | --- | --- | --- | --- | --- | --- |
|  | | 1 | 2 | 3 | 4 | 5 |
| a | Faculty knowledge and dedication to teaching is very high |  |  |  |  |  |
| b | Faculty always show up to conduct teaching sessions when scheduled |  |  |  |  |  |
| c | Faculty private practice responsibilities interferes with teaching responsibilities |  |  |  |  |  |
| d | Faculty keep up to date in the latest developments in their field |  |  |  |  |  |
| e | Faculty are generally good teachers with a strong interest in helping us learn |  |  |  |  |  |
| f | The basic sciences curricula were generally well organized to help me learn |  |  |  |  |  |
| g | Clinical rotations are well generally organized to help me learn the topic thoroughly |  |  |  |  |  |
| h | Access to current textbooks and journals is generally good |  |  |  |  |  |

1. How would you compare the quality of your medical training in Iraq to what you know of medical schools in other countries in the region? *(check only one)*

*A Much better B About the same C Worse D Much worse E Do not know*

1. What areas of your medical education do you think were particularly good?
2. What of your medical education do you think were particularly poor?
3. Do you have any suggestions to improve medical education in Iraq?

**B. Future plans**

1. Which of the following statements best describes your future educational plans? ***Circle only one***

| a | I am still undecided about my future plans |
| --- | --- |
| b | On graduation I will not seek further training, and become a general practitioner |
| c | Family Medicine |
| d | Anesthesia |
| e | Pediatrics |
| f | OBS/GYN |
| g | Surgery or the surgical specialties (ENT, urology, neurosurgery etc) |
| h | Internal medicine or the medical specialties (cardiology, nephrology, etc) |
| i | Radiology |
| j | Dermatology |
| k | Public Health or Community Medicine |
| l | Pathology |
| m | Psychiatry |
| n | Other (specify)_______________________________________ |

1. Which statement best describes your plans at this time for further training after graduation?

| a | I have no plans at this time |
| --- | --- |
| b | I am trying to decide, and have some idea |
| c | I have clear ideas about what I will do on qualification |

1. If you have some plans (b-c) even if not completed, where will you do this training?

| a | I will definitely do my specialty train in Iraq |
| --- | --- |
| b | I am actively looking to do my specialty training outside Iraq, but no specific plans |
| c | I have definitely and specific plans to do my specialty training outside Iraq |

1. Where do you see yourself working after you complete all of your training?

| **1** = definitely not **2** = possibly  **3** = very likely | | **1** | **2** | **3** |
| --- | --- | --- | --- | --- |
| a | Baghdad |  |  |  |
| b | Iraq, but not Baghdad |  |  |  |
| c | Other Middle East countries including the Persian Gulf |  |  |  |
| d | Europe |  |  |  |
| e | Australia or New Zealand |  |  |  |
| f | Canada |  |  |  |
| g | United States |  |  |  |

1. How often do you think of leaving Iraq after your graduation from medical school? *(circle one)*

*A Not at all B Occasionally C Frequently D All the time*

1. What would be your biggest motivators for living in Iraq after you complete all of training?

| 1 = most important 2 = somewhat important 3 = not very important 4 = least important | | **1** | **2** | **3** | **4** |
| --- | --- | --- | --- | --- | --- |
| a | I am familiar with the health care system in Iraq |  |  |  |  |
| b | To be with my family and friends |  |  |  |  |
| c | This is my country and I feel responsibilities |  |  |  |  |
| d | The positions I can get here are better than I can get outside Iraq |  |  |  |  |
| e | The personal life style in Iraq is what I like |  |  |  |  |

1. How would you rate your motivators for going outside of Iraq?

| **1** = most important **2** = somewhat important  **3** = not very important 4 = least important | | 1 | 2 | 3 | 4 |
| --- | --- | --- | --- | --- | --- |
| a | Seek advanced training |  |  |  |  |
| b | Better professional opportunities |  |  |  |  |
| c | Be with family: |  |  |  |  |
| d | Better pay and working conditions |  |  |  |  |
| e | Avoid war and conflict |  |  |  |  |
| f | Better personal lifestyle |  |  |  |  |

1. In the past 3 years do you think the desirability to practice medicine as a young doctor in Iraq has-- *(circle one)*

*A Gotten better B stayed the same C Gotten worse D not sure*

1. Do you think the professional satisfaction that doctors get from the practice of medicine in Iraq has--

*A Gotten better B stayed the same C Gotten worse D not sure*

1. In the past 3 years to you think the potential of living a satisfying personal life while practicing medicine in Iraq has— *(circle one)*

*A Gotten better B stayed the same C Gotten worse D not sure*

**C. In the next section are some questions about your opinions of health care in Iraq now**

1. How would you rate health care in Iraq today?

| **1** = Excellent **2** = Good  **3** = Fair 4 = Poor 5= Don’t know | | 1 | 2 | 3 | 4 | 5 |
| --- | --- | --- | --- | --- | --- | --- |
| a | The quality of health care received in hospitals |  |  |  |  |  |
| b | The quality of health care received in Primary Health Care Clinics (PHCCs) |  |  |  |  |  |
| c | The quality of health care received in private clinics |  |  |  |  |  |
| d | The availability of medicines and supplies when needed |  |  |  |  |  |
| e | The availability of laboratory tests when needed |  |  |  |  |  |
| d | The attitude and concern of doctors workers for patients |  |  |  |  |  |
| e | The attitude and concern of other health care workers for patients |  |  |  |  |  |
| f | The attitude of patients and their family toward doctors |  |  |  |  |  |
| g | Salary and income for doctors |  |  |  |  |  |
| h | Working conditions for doctors |  |  |  |  |  |
| i | Safety and security for doctors |  |  |  |  |  |

1. How would you rate the availability of the following components of health care in urban areas?

|  | | | | |  |
| --- | --- | --- | --- | --- | --- |
| **1** = More than enough **2** = Adequate  **3** = Shortage 4 = Severe shortage | | 1 | 2 | 3 | 4 |
| a | The number of hospitals |  |  |  |  |
| b | The number of Primary Health Care Clinics (PHCCs) |  |  |  |  |
| c. | The number of private clinics |  |  |  |  |
| d | The number of nurses in hospitals and clinics |  |  |  |  |
| e | The number of technicians (laboratory, x-ray, pharmacy etc) |  |  |  |  |
| f | The availability of medicines and supplies when needed |  |  |  |  |
| g | The availability of laboratory tests when needed |  |  |  |  |

**D. The effects of conflict and insecurity on your professional choices**

1. To what extent are your career choices affected by the political and conflicts in Iraq *(circle one)*

*A Very much B Somewhat C A little bit D Not at all*

1. Did any immediate family (parents or siblings) members die in the war that started in 2003?

*A Yes B No*

A. If so, how many? _______

B. How many immediate family members were seriously injured? _______

1. Did any of your medical school classmates or faculty die in the war that started in 2003? Y N

A. If so, how many? _______

1. Do you know of any medical school faculty members who left Iraq during your medical school years? If so, how many do you know of? _______

*A Yes B No*
